# Integrated metabolomic and transcriptomic analyses reveal tissue-specific distribution and gene-metabolite associations of quality-related metabolites in the Tibetan Plateau turnip landrace “Yushuyuangen” (*Brassica rapa* ssp. *rapa*)

**DOI:** 10.3389/fnut.2026.1980490

**Published:** 2026-10-13

**Authors:** Caixia Li, Ruxue Qian, Quanhui Li, Yanjing Ren

**Affiliations:** 1 Laboratory of Research and Utilization of Germplasm Resources in Qinghai-Tibet Plateau, Academy of Agriculture and Forestry Sciences of Qinghai Province, Qinghai University, Xining, Qinghai, China; 2 Key Laboratory of Germplasm Resources Protection and Genetic Improvement of the Qinghai-Tibet Plateau in Ministry of Agriculture and Rural, Xining, Qinghai, China

**Keywords:** amino acid metabolism, flavonoid metabolism, phenylpropanoid metabolism, sugar metabolism, Yushuyuangen turnip

## Abstract

Yushuyuangen (*Brassica rapa* ssp. *rapa*) is a characteristic turnip landrace cultivated on the Qinghai–Tibet Plateau and valued for its nutritional and flavor qualities. However, the tissue-specific distribution of quality-related metabolites and the gene-expression patterns associated with their accumulation remain poorly understood. Here, metabolomics and transcriptomics were integrated with weighted gene co-expression network analysis (WGCNA) to investigate three root tissues in two varieties, YPW (purple-white) and YGW (green-white): the upper colored peel (purple in YPW and green in YGW), lower white peel, and root flesh. A total of 775 metabolites were identified, showing clear tissue-dependent metabolic differentiation. Sugar-phosphate intermediates and aromatic/aspartate-family amino acids accumulated mainly in root flesh (glucose-6-phosphate, fructose-6-phosphate, phenylalanine, and homoserine), whereas branched-chain amino acids were abundant in colored peel (valine and leucine). Purple peel accumulated higher levels of *p-*coumaric acid and ferulic acid, while flavonoids differed markedly between purple and green peel, represented by phloretin and diosmetin in purple peel and trifolin and kaempferol-7-*O*-glucoside in green peel. Integrated WGCNA and correlation analyses further identified tissue-associated relationships among metabolites, structural genes, and transcription factors. Four candidate transcription factor–structural gene–metabolite associations were highlighted: *NAC22*–*F16P2.1*–D-fructose-6-phosphate, *bHLH65*/*ARFD4*–*TRBP2.2*–L-tryptophan, *MYB62*/*AGL63*–*PAL1.2*/*C4H.2*–*p-*coumaric acid, and *MYB62*–*FLS1*/*F3′H1.2*–trifolin/kaempferol-7-*O*-glucoside. These associations were inferred from co-expression and correlation patterns and represent candidate relationships for further functional validation.

## 1 Introduction

Turnip (*Brassica rapa* ssp. *rapa*) is an important root vegetable in the Brassicaceae family and has a long history of cultivation and consumption, particularly in Europe, Central Asia, and East Asia (1). Turnip is rich in various nutrients and bioactive compounds (2, 3), including organic acids, sugars, amino acids, glucosinolates (GSLs), phenolic acids, flavonoids and ascorbic acid (4, 5). Free sugars and amino acids are important contributors to turnip flavor, influencing sweetness as well as other taste attributes, including bitterness and aftertaste (6, 7). Phenylpropanoid metabolism produces diverse phenolic acids and flavonoids that contribute to tissue coloration, taste-related properties, and the nutritional quality of turnip (8). In addition to contributing to nutritional and sensory quality, bioactive compounds in turnip have been investigated for their potential antioxidant and anti-inflammatory activities (2, 9, 10), highlighting their potential as ingredients in plant-based functional foods.

Turnip root color varies widely among germplasm accessions, which is associated with differences in secondary metabolite accumulation and biosynthetic gene expression. For example, purple turnip peel accumulates higher levels of anthocyanins than green turnip peel, together with differential expression of anthocyanin biosynthetic genes such as *DFR*, *ANS*, and *UFGT* (11). Similarly, yellow- and white-fleshed turnips differ markedly in carotenoid accumulation, and these differences are associated with differential expression of *PSY* (12). These findings indicate that tissue coloration partly reflects variation in secondary metabolism; however, metabolic differentiation among storage-root tissues extends beyond pigmentation to a broader range of quality-related metabolites. In potato tubers, the flesh preferentially accumulates primary metabolites, including sugars, amino acids and vitamins, whereas the skin is enriched in secondary metabolites such as glycoalkaloids and flavonoids, accompanied by distinct pathway enrichment and gene expression patterns (13). Similar tissue-specific differentiation has been observed in radish, with flavonoids and phenolic acids accumulating abundantly in red skin and red flesh and several glucosinolates preferentially accumulating in green skin, accompanied by tissue-specific expression of associated structural genes and transcription factors (14, 15).

Multi-omics studies have revealed regulation of primary and secondary metabolism in root and tuber crops. In sweet potato storage roots, genes involved in sucrose metabolism, sugar transport, and starch biosynthesis, together with transcription factors such as *SRF1* and *IbbHLH49*, contribute to carbohydrate accumulation (16–18). Comparative analyses of differently colored radish skin and flesh identified tissue-specific flavonoid and glucosinolate profiles associated with differential expression of *CHS*, *CHI*, *F3H*, *FLS*, *F3′H*, *DFR*, and *ANS*, as well as several MYB and bHLH transcription factors (14, 15). In turnip root skin, differences in anthocyanin accumulation between purple and green tissues were accompanied by contrasting expression of *FLS*, *DFR*, *ANS*, and *UFGT* and several candidate MYB, bHLH, and WRKY transcription factors (11). Tissue-dependent anthocyanin accumulation has also been reported in potato tubers, where *StWRKY44* was shown to activate multiple structural genes involved in phenylpropanoid and flavonoid biosynthesis (19).

Previous studies of turnip have largely focused on specific colored tissues or individual metabolite classes, such as anthocyanins in purple and green peel or carotenoids in yellow and white root flesh, whereas the overall metabolic differences among colored peel, white peel, and root flesh have not been systematically characterized. In particular, little is known about how primary and secondary metabolites are partitioned among these storage-root tissues in high-altitude *Brassica rapa* landraces, or how such tissue-specific metabolic patterns are associated with structural gene expression and candidate transcription factors. Therefore, integrated metabolomic and transcriptomic analyses are needed to characterize the metabolic composition of different Yushuyuangen root tissues and identify the molecular components associated with their tissue-specific metabolic differentiation.

In this study, green–white and purple–white Yushuyuangen varieties were used to investigate metabolic differentiation among colored peel, white peel, and root flesh. We hypothesized that these tissues exhibit distinct primary and secondary metabolic profiles, with both shared and variety-associated patterns accompanied by differential expression of pathway-related structural genes and transcription factors. To test this hypothesis, integrated metabolomic and transcriptomic analyses, together with WGCNA and transcription factor–gene–metabolite association analysis, were performed to characterize tissue-specific metabolic differences and identify candidate genes and transcription factors associated with sugar, amino acid, phenylpropanoid-phenolic acid, and phenylpropanoid-flavonoid metabolism.

## 2 Materials and methods

### 2.1 Plant material

Different tissues of local varieties Yushuyuangen, purple-white type (YPW), and green-white type (YGW) were collected in Qianduo Village, Xiangda Town, Nangqian County, Yushu Tibetan Autonomous Prefecture, Qinghai Province during the local harvest in mid-October 2023, when the storage roots had reached commercial maturity. The purple-white variety was further divided into three tissues: purple peel (YPW-PP), white peel (YPW-WP), and root flesh (YPW-SR); the green-white variety was divided into green peel (YGW-GP), white peel (YGW-WP), and root flesh (YGW-SR) (Supplementary Figure S1). For both YPW and YGW, colored peel, white peel, and root flesh were each represented by three biological replicates, with each replicate obtained from a single individual plant. No pooling of biological samples was performed before or after extraction. Thus, a total of 18 samples were collected for subsequent analyses.

### 2.2 Extraction of samples for UPLC-MS/MS analysis

Sample extraction, metabolite separation, mass spectrometric detection, and metabolite quantification were carried out by MetWare Biotechnology Co., Ltd. (Wuhan, China). Briefly, freeze-dried samples were extracted with 70% methanol and subjected to UPLC–ESI–MS/MS analysis. Metabolites were identified based on the MetWare database (MWDB) and MS/MS information and relatively quantified using multiple reaction monitoring (MRM) (20).

### 2.3 RNA-sequencing analysis

The extraction of total RNA and RNA-sequencing analysis of various Yushuyuangen samples were carried out by a commercial service company (MetWare Biotechnology, Wuhan, China) based on previously described protocols (21) with minor modifications. In brief, plant samples were processed using the CTAB-PBIOZOL and ethanol precipitation method for RNA extraction. Oligo (dT) magnetic beads were employed to enrich mRNA, which was subsequently fragmented and reverse-transcribed into strand-specific cDNA (22). The cDNA underwent end repair, A-addition, adapter ligation, purification, and PCR amplification. After sequencing and quality control, the clean reads were mapped to the *Brassica rapa* genome v3.0 (23).

### 2.4 Data processing and analysis

For metabolomic data, orthogonal partial least squares discriminant analysis (OPLS-DA) was performed to further evaluate metabolic differences among sample groups. The fitting and predictive performance of the OPLS-DA models was assessed using R^2^X, R^2^Y, and Q^2^, with all nine models showing R^2^X > 0.45, R^2^Y > 0.99, and Q^2^ > 0.61; model robustness and potential overfitting were further evaluated by 200 permutation tests (Supplementary Figure S2). Differentially accumulated metabolites (DAMs) were identified based on the variable importance in projection (VIP) values derived from the OPLS-DA models and fold change (FC), with VIP ≥ 1.0 and fold change ≥ 2.0 or ≤ 0.5 as the screening criteria. DAMs were annotated against the Kyoto Encyclopedia of Genes and Genomes (KEGG) database, and enrichment results were evaluated. For transcriptomic data, differential expression analysis was performed using DESeq2, and *p* values were adjusted for multiple hypothesis testing. Differentially expressed genes (DEGs) were identified using |log₂Fold Change| ≥ 1 and false discovery rate (FDR) < 0.05 as the screening criteria. DEGs were also subjected to KEGG and GO pathway annotation and enrichment analysis, and pathways with q-values < 0.05 after multiple-testing correction were considered significantly enriched.

### 2.5 WGCNA analysis

Based on the nine-quadrant plots generated from the integrated metabolomic and transcriptomic analysis, gene–metabolite correlations were filtered using thresholds of |*r*| > 0.8 and *p* < 0.05. The genes retained after correlation filtering were then combined, and duplicate genes were removed. The FPKM expression matrix of the remaining non-redundant genes was subsequently used for weighted gene co-expression network analysis (WGCNA). The co-expression network was constructed using the WGCNA package in R. Based on the scale-free topology criterion, the soft-thresholding power was set to 18, and the adjacency matrix was transformed into a topological overlap matrix (TOM). Co-expression modules were identified using the dynamic tree cut algorithm with a minimum module size of 50 genes, and highly similar modules were subsequently merged at a cut height of 0.25. Pearson correlation analysis was then performed between module eigengenes and metabolites, including sugars, amino acids, phenolic acids, and flavonoids, to identify metabolite-associated co-expression modules.

Based on the metabolite-associated modules identified by WGCNA, structural genes involved in the corresponding metabolic pathways and transcription factors within these modules were further selected. Pairwise Pearson correlation analyses were performed among metabolites, structural genes, and transcription factors. Metabolite–gene–transcription factor combinations for which all three pairwise correlations satisfied |*r*| > 0.8 were retained for subsequent network construction. The resulting metabolite–gene–transcription factor networks were visualized using Cytoscape v3.7.2 (24).

## 3 Results

### 3.1 Comparative metabolomic analysis in Yushuyuangen

To further characterize metabolic differences among Yushuyuangen tissues and varieties, metabolomic profiling was performed using UPLC–MS/MS. The analytical platform showed good reproducibility, as more than 75% of metabolites in the quality-control samples exhibited coefficients of variation below 0.3 and the total ion chromatograms showed a high degree of overlap (Supplementary Figure S3). A total of 775 metabolites were detected, including 128 lipids, 122 phenolic acids, 87 flavonoids, 85 amino acids and derivatives, 65 alkaloids, 60 organic acids, 47 nucleotides and derivatives, 31 lignans and coumarins, 13 terpenoids, 4 quinones and 1 tannin. The remaining 132 metabolites were grouped as “others,” comprising 58 saccharides and alcohols, 38 glucosinolates, 19 vitamins, and 17 unclassified metabolites (Figure 1A; Supplementary Table S1). PCA revealed clear differences in metabolite profiles among tissues, with PC1 and PC2 explaining 30.22 and 16.39% of the total variance, respectively (Figure 1B). The three tissues within each variety were well separated, whereas corresponding tissues of YPW and YGW generally clustered more closely. Among the corresponding tissues, the colored peels (YPW-PP and YGW-GP) showed the greatest separation.

**Figure 1:**
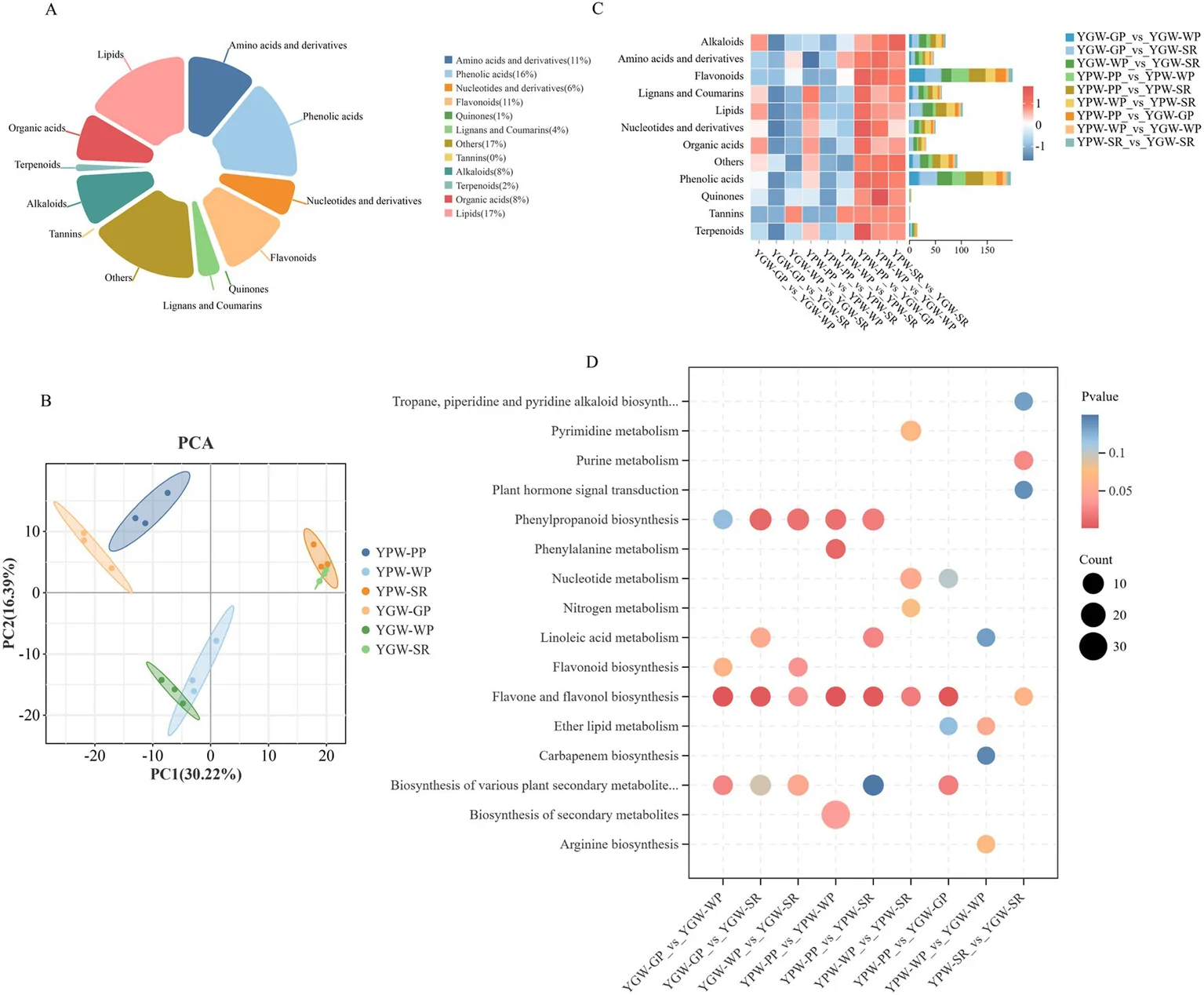
Comprehensive metabolomic analysis of different tissues of Yushuyuangen. **(A)** Classification and relative proportion of identified metabolites. **(B)** Principal component analysis (PCA) illustrating metabolic variation among tissues and varieties. **(C)** Differentially accumulated metabolites (DAMs) identified across tissues and varieties. **(D)** KEGG pathway enrichment analysis of DAMs.

Differentially accumulated metabolites (DAMs) were further identified among tissues within each variety (Figure 1C). In YPW, 200, 293, and 250 DAMs were identified in YPW-PP vs. YPW-WP, YPW-PP vs. YPW-SR, and YPW-WP vs. YPW-SR, respectively. In all three comparisons, most DAMs showed higher accumulation levels in the former tissue, accounting for 157 (78.5%), 263 (89.8%), and 205 (82.0%) DAMs, respectively. Phenolic acids and flavonoids were among the major DAM classes. Several representative metabolites showed tissue-specific detection patterns. In the YPW-PP vs. YPW-WP comparison, kaempferol-3-*O*-(2″′-malonyl)sophoroside-7-*O*-rutinoside, protocatechuic acid methyl ester, protocatechuic acid, luteolin-7-*O*-(2″″-*O*-rhamnosyl)sophorotrioside, and 2,3-dihydroxybenzoic acid were detected in YPW-PP but not in YPW-WP. In addition, N-(4-aminobutyl)benzamide, naringenin-7-*O*-(6″-malonyl)glucoside, quercetin-3-*O*-(2″-*O*-acetyl)glucuronide, and 6-*O*-galloyl-β-D-glucose were detected in both YPW-PP and YPW-WP but not in YPW-SR (Supplementary Figures S4A–C).

A similar overall pattern was observed in YGW. A total of 164, 308, and 276 DAMs were identified in YGW-GP vs. YGW-WP, YGW-GP vs. YGW-SR, and YGW-WP vs. YGW-SR, respectively. In all three comparisons, most DAMs showed higher accumulation levels in the former tissue, accounting for 139 (84.8%), 284 (92.2%), and 232 (84.1%) DAMs, respectively. In the YGW-GP vs. YGW-WP comparison, quercetin-3-*O*-gentiotetroside, *p-*coumaric acid-4-*O*-glucoside, kaempferol-3-*O*-(6″-feruloyl)glucosyl-(1 → 4)-galactoside, and eriodictyol-3′-*O*-glucoside were detected in YGW-GP but not in YGW-WP. In addition, 7S,8S-DiHODE [(9Z,12Z)-(7S,8S)-dihydroxyoctadeca-9,12-dienoic acid], naringenin-7-*O*-(6″-malonyl)glucoside, 6-*O*-galloyl-β-D-glucose, and quercetin-3-*O*-(2″-*O*-acetyl)glucuronide were detected in both YGW-GP and YGW-WP but not in YGW-SR. Varietal comparisons identified 136, 64, and 70 DAMs in YPW-PP vs. YGW-GP, YPW-WP vs. YGW-WP, and YPW-SR vs. YGW-SR, respectively. The colored-peel comparison contained the largest number of DAMs, of which 97 showed higher accumulation levels in YGW-GP (Supplementary Figures S4E,F).

KEGG enrichment analysis was performed to characterize the metabolic pathways associated with the DAMs (Figure 1D). In comparisons between colored and white peel tissues, DAMs in both YGW-GP vs. YGW-WP and YPW-PP vs. YPW-WP were enriched in phenylpropanoid biosynthesis and flavone and flavonol biosynthesis. Flavonoid biosynthesis was additionally enriched in YGW-GP vs. YGW-WP, whereas phenylalanine metabolism was enriched in YPW-PP vs. YPW-WP. In comparisons between peel and root flesh, phenylpropanoid biosynthesis was enriched in three comparisons, while flavone and flavonol biosynthesis was enriched in all four comparisons. Linoleic acid metabolism was enriched in YGW-GP vs. YGW-SR and YPW-PP vs. YPW-SR, whereas YPW-WP vs. YPW-SR additionally showed enrichment in pyrimidine metabolism, nucleotide metabolism, and nitrogen metabolism. Among the inter-varietal comparisons, YPW-PP vs. YGW-GP was enriched in flavone and flavonol biosynthesis and biosynthesis of various plant secondary metabolites, YPW-WP vs. YGW-WP in ether lipid metabolism and arginine biosynthesis, and YPW-SR vs. YGW-SR in purine metabolism and flavone and flavonol biosynthesis.

### 3.2 Comparative transcriptomic analysis identifies tissue-associated expression patterns in Yushuyuangen

To further investigate transcriptional differences among Yushuyuangen tissues and varieties, RNA sequencing was performed using the same 18 samples subjected to metabolomic profiling. After quality filtering, a total of 118.58 Gb of clean data were obtained, with an average Q30 value of 92.51% and a mean GC content of 47.03% (Supplementary Table S2). The clean reads were mapped to the *Brassica rapa* reference genome and used for subsequent differential expression analysis. Differential expression analysis revealed substantial differences in gene expression among tissues (Figure 2A). In YPW, 514, 2,516, and 2,774 DEGs were identified in YPW-PP vs. YPW-WP, YPW-PP vs. YPW-SR, and YPW-WP vs. YPW-SR, respectively. In YGW, 1,012, 3,245, and 3,475 DEGs were identified in YGW-GP vs. YGW-WP, YGW-GP vs. YGW-SR, and YGW-WP vs. YGW-SR, respectively. In both varieties, more DEGs were identified between peel and root flesh than between the colored and white peel tissues. Inter-varietal comparisons identified 1,316, 1,632, and 1,724 DEGs in YPW-PP vs. YGW-GP, YPW-WP vs. YGW-WP, and YPW-SR vs. YGW-SR, respectively (Figure 2A). Among the corresponding tissues, the root-flesh comparison contained the largest number of DEGs.

**Figure 2:**
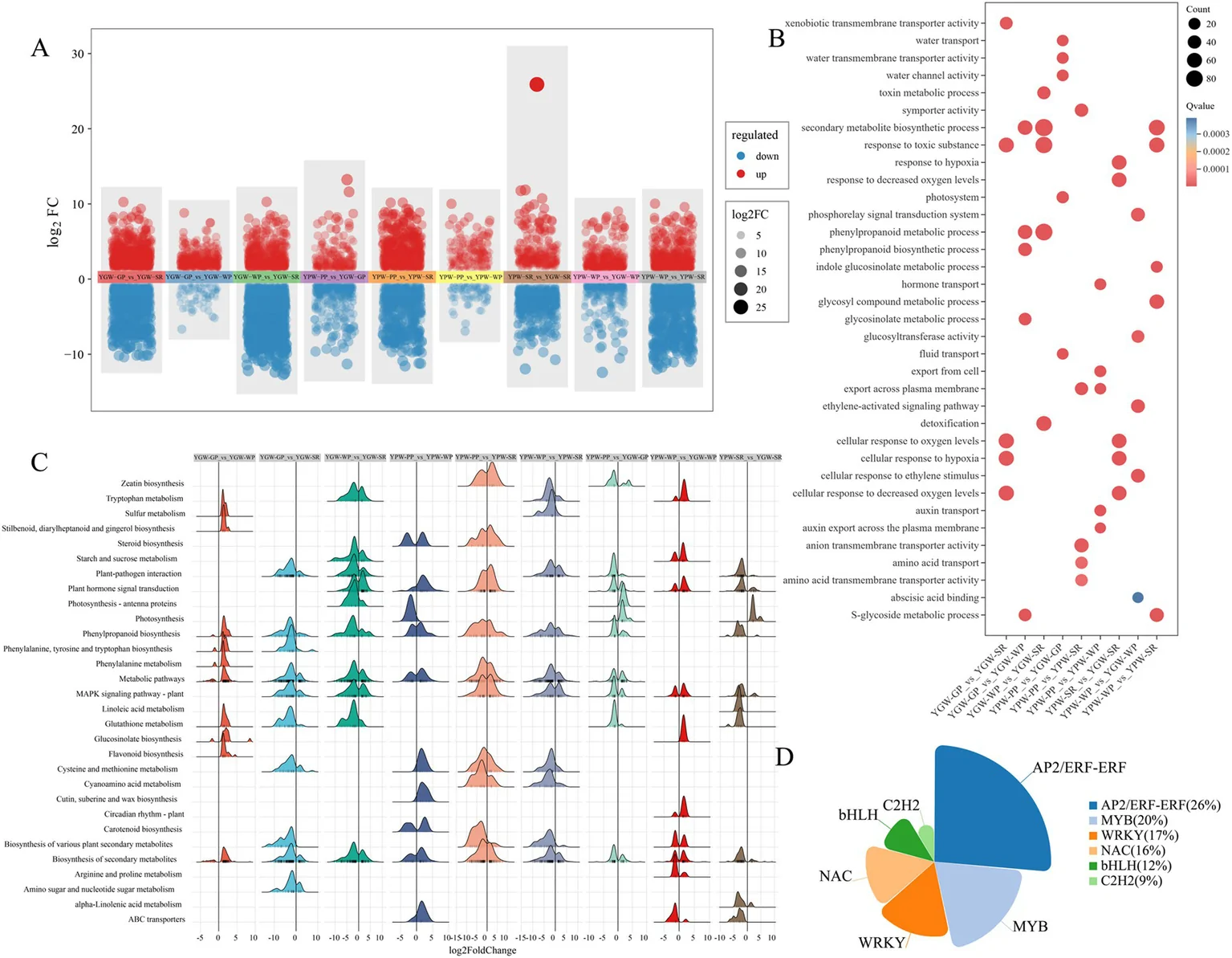
Transcriptomic profiling of different tissues of Yushuyuangen. **(A)** Distribution of log₂ fold changes for upregulated and downregulated genes across the nine pairwise comparisons. **(B)** Gene Ontology (GO) enrichment analysis of DEGs. **(C)** Ridgeline density plots showing the log₂ fold-change distributions of DEGs in selected KEGG pathways across the nine comparisons. **(D)** Distribution of the major families of differentially expressed transcription factors.

GO enrichment analysis was performed to characterize the biological functions associated with the DEGs (Figure 2B). In comparisons between colored and white peel tissues, DEGs in YGW-GP vs. YGW-WP were significantly enriched in phenylpropanoid biosynthetic process and phenylpropanoid metabolic process, whereas those in YPW-PP vs. YPW-WP were significantly enriched in hormone transport, auxin transport, and auxin export across the plasma membrane. In comparisons between peel and root flesh, DEGs in YGW-GP vs. YGW-SR were significantly enriched in xenobiotic transmembrane transporter activity, response to toxic substance, response to hypoxia, and response to decreased oxygen levels, whereas those in YGW-WP vs. YGW-SR were significantly enriched in toxin metabolic process, phenylpropanoid metabolic process, and detoxification. In YPW, DEGs in YPW-PP vs. YPW-SR were significantly enriched in amino acid transport, amino acid transmembrane transporter activity, and anion transmembrane transporter activity, whereas those in YPW-WP vs. YPW-SR were significantly enriched in response to toxic substance, indole glucosinolate metabolic process, and S-glycoside metabolic process. Among the inter-varietal comparisons, DEGs in YPW-PP vs. YGW-GP were significantly enriched in water transport, water transmembrane transporter activity, water channel activity, and photosystem; those in YPW-WP vs. YGW-WP were enriched in phosphorelay signal transduction system, glucosinolate metabolic process, and ethylene-activated signaling pathway; and those in YPW-SR vs. YGW-SR were significantly enriched in response to hypoxia, cellular response to hypoxia, and cellular response to decreased oxygen levels.

KEGG enrichment analysis was performed to characterize the pathways associated with the DEGs, and the log₂ fold-change distributions of DEGs in selected pathways were visualized using ridgeline density plots (Figure 2C). In comparisons between colored and white peel tissues, phenylpropanoid biosynthesis and flavonoid biosynthesis were enriched in both varieties. In YGW-GP vs. YGW-WP, these pathways, together with phenylalanine metabolism and glutathione metabolism, were significantly enriched, whereas alpha-linolenic acid metabolism was enriched in YPW-PP vs. YPW-WP. In comparisons between peel and root flesh, phenylpropanoid biosynthesis and biosynthesis of secondary metabolites were enriched across all comparisons. In YGW-GP vs. YGW-SR, additional enriched pathways included phenylalanine metabolism, linoleic acid metabolism, and glutathione metabolism, whereas YGW-WP vs. YGW-SR showed significant enrichment in starch and sucrose metabolism. In YPW, YPW-PP vs. YPW-SR additionally showed enrichment in phenylalanine metabolism, cysteine and methionine metabolism, and flavonoid biosynthesis, whereas YPW-WP vs. YPW-SR showed significant enrichment in tryptophan metabolism, sulfur metabolism, and cysteine and methionine metabolism. Among the inter-varietal comparisons, several pathways were represented in more than one corresponding-tissue comparison, including plant hormone signal transduction, phenylpropanoid biosynthesis, MAPK signaling pathway-plant, and biosynthesis of secondary metabolites. In YPW-PP vs. YGW-GP, other representative pathways included starch and sucrose metabolism, photosynthesis-antenna proteins, photosynthesis, and glutathione metabolism. In YPW-WP vs. YGW-WP, other representative pathways included tryptophan metabolism, glucosinolate biosynthesis, and arginine and proline metabolism. In YPW-SR vs. YGW-SR, other representative pathways included plant-pathogen interaction, linoleic acid metabolism, alpha-linolenic acid metabolism, and ABC transporters.

Transcription factors (TFs) play important regulatory roles in plant growth, development, and maturation. A total of 836 differentially expressed TFs were identified and classified into 52 TF families. Among them, AP2/ERF-ERF (114 genes), MYB (87), WRKY (73), NAC (67), bHLH (53), and C2H2 (37) were the most abundant TF families (Figure 2D).

### 3.3 Associated transcriptome-metabolome analysis comparing Yushuyuangen for quality traits

To comprehensively characterize the molecular basis underlying taste and nutritional quality differences between YGW and YPW, an integrated metabolomic and transcriptomic analysis was performed. Integrated KEGG pathway enrichment analysis identified multiple primary and secondary metabolic pathways across tissue and varietal comparisons (Supplementary Figure S5). Phenylpropanoid biosynthesis, flavone and flavonol biosynthesis, and biosynthesis of secondary metabolites were enriched in both the metabolomic and transcriptomic datasets, particularly in peel-related comparisons. Primary metabolic pathways related to amino acid and sugar metabolism were also enriched, indicating that both primary and secondary metabolism contributed to the metabolic differences among tissues.

Based on the nine-quadrant plots generated from the integrated metabolomic and transcriptomic analysis, gene–metabolite correlations were filtered using thresholds of *|r*| > 0.8 and *p* < 0.05. After removal of duplicated gene entries, 11,956 genes were retained for WGCNA. Of these, 11,942 genes were assigned to 20 co-expression modules, while the remaining 14 genes were grouped into the grey module as unassigned genes (Supplementary Figure S6; Figure 3A). Module sizes ranged from 92 genes in the royalblue module to 2,120 genes in the turquoise module.

**Figure 3:**
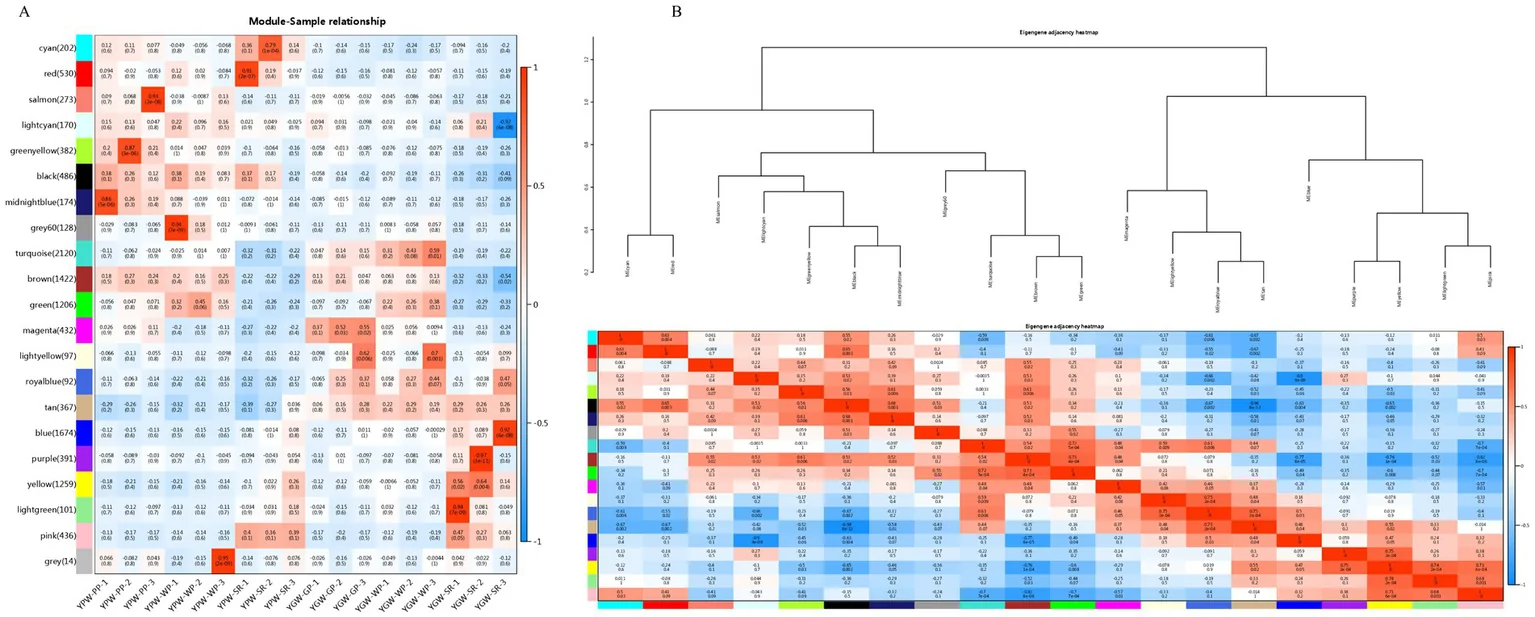
Weighted gene co-expression network analysis (WGCNA) of Yushuyuangen transcriptome data. **(A)** Module–sample relationship heatmap showing the correlations between gene co-expression modules and different tissues of Yushuyuangen. Colors represent the strength and direction of correlations between module eigengenes and sample groups. **(B)** Hierarchical clustering dendrogram and eigengene adjacency heatmap illustrating the relationships among module eigengenes.

Module–sample correlation analysis revealed distinct correlation patterns between co-expression modules and Yushuyuangen samples (Figure 3A). For example, the magenta module showed positive correlations with the three YGW green-peel replicates, with correlation coefficients of *r* = 0.37 (*p* = 0.13), 0.52 (*p* = 0.027), and 0.55 (*p* = 0.018) for YGW-GP-1, YGW-GP-2, and YGW-GP-3, respectively. Other modules also showed sample-specific correlation patterns, and the complete correlation coefficients and corresponding *p* values are presented in Figure 3A. These module–sample relationships were subsequently used to support the identification of structural genes and transcription factors associated with quality-related metabolic pathways. Module eigengene adjacency analysis further showed that several modules exhibited similar eigengene profiles and clustered closely together, whereas others displayed relatively distinct expression patterns (Figure 3B). These co-expression relationships provided a basis for the subsequent identification of structural genes and transcription factors associated with quality-related metabolic pathways.

### 3.4 Integrated analysis of metabolites and gene expression in sugar metabolism of Yushuyuangen

Eight DAMs and 40 DEGs associated with glycolysis, gluconeogenesis, and related metabolic pathways were integrated to reconstruct a sugar metabolism network (Figure 4). Root flesh was characterized by higher accumulation of most sugar metabolism intermediates, with both shared and variety-dependent patterns. Glucose-1-phosphate, D-glucose-6-phosphate, D-fructose-6-phosphate, and D-erythrose-4-phosphate accumulated higher levels in the root flesh of both varieties. Glucose-1-phosphate and D-glucose-6-phosphate were negatively correlated with genes encoding glucose-6-phosphate isomerase (*G6PIP.1* and *G6PIP.2*) and transketolase (*TKTC2*), which showed higher expression in white peel, particularly YGW-WP for *TKTC2*. D-fructose-6-phosphate was likewise negatively correlated with *G6PIP.1* (*r* = −0.9530, *p* < 0.01) and *G6PIP.2* (*r* = −0.9058, *p* < 0.01), but positively correlated with genes encoding ATP-dependent 6-phosphofructokinase (*PFKA7*), fructose-1,6-bisphosphatase (*F16P2.1*), and fructose-bisphosphate aldolase (*ALFP2.3*), all of which showed higher expression in YGW-SR. D-erythrose-4-phosphate was negatively correlated with *G6PIP.1*, *G6PIP.2*, *TKTC2*, and genes encoding alcohol dehydrogenase-like 6 (*ADHL6*) and transaldolase (*TALDO*); the latter two showed higher expression in YGW-WP and YPW-WP, respectively. Within root flesh, D-fructose-1,6-bisphosphate and phosphoenolpyruvate showed their highest accumulation in YPW-SR, whereas D-sedoheptulose-7-phosphate accumulated at higher levels in the root flesh of both varieties, particularly in YGW-SR. The latter was negatively correlated with genes encoding hexokinase (*HXK3.1* and *HXK3.2*) and with *G6PIP.1*, all of which showed low expression in YGW-SR. In contrast, dihydroxyacetone phosphate (DHAP) showed higher accumulation in purple peel (YPW-PP) and green peel (YGW-GP), particularly the latter (Figure 4).

**Figure 4:**
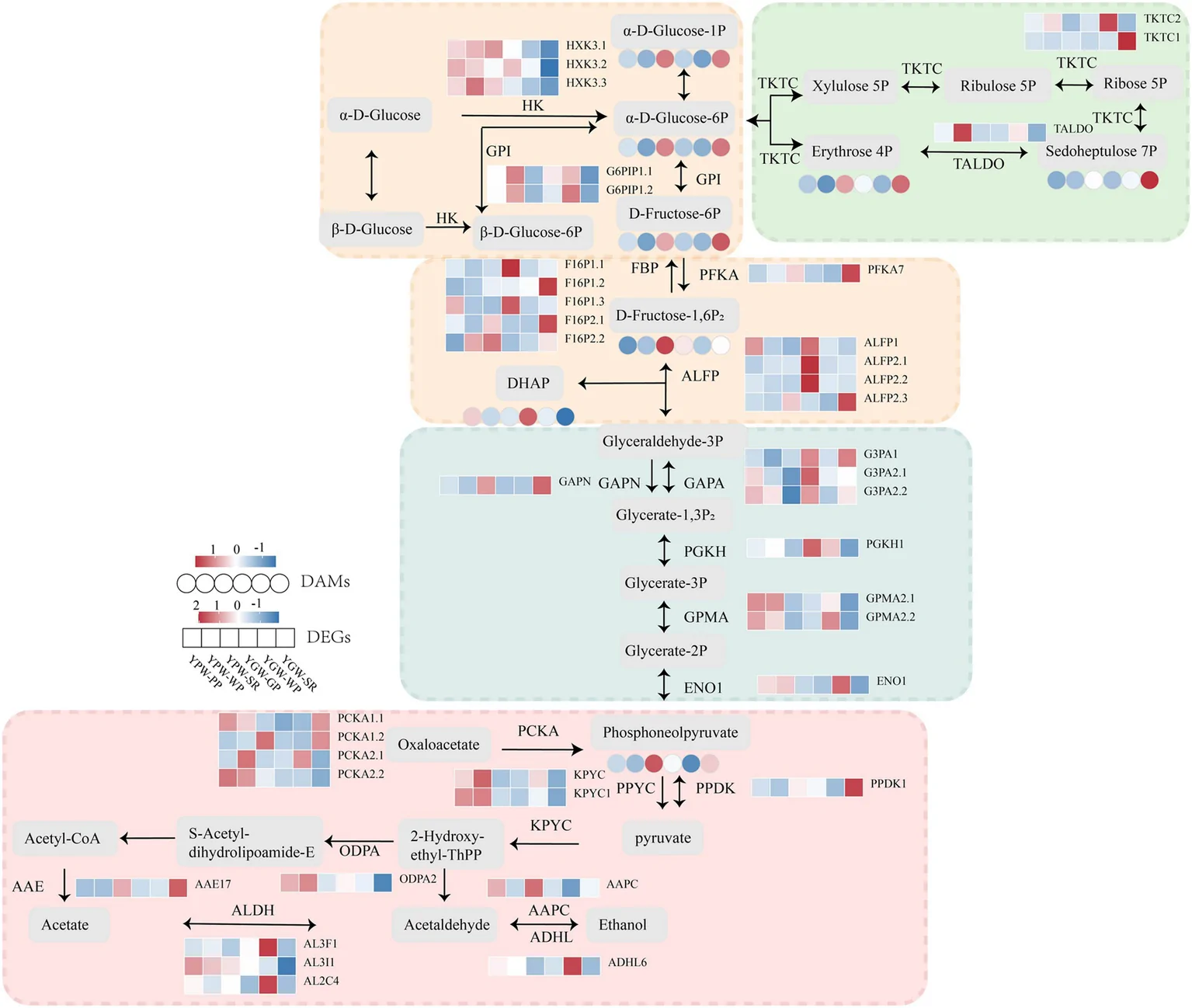
Integrated reconstruction of glycolysis and gluconeogenesis pathways in Yushuyuangen. Differentially expressed genes (DEGs) and differentially accumulated metabolites (DAMs) were mapped onto the metabolic pathways. Red and blue represent up- and down-regulation, respectively.

WGCNA identified four modules associated with these metabolic patterns (Figure 5A). The yellow and blue modules were positively correlated with glucose-1-phosphate, D-glucose-6-phosphate, D-fructose-6-phosphate, D-sedoheptulose-7-phosphate, and D-erythrose-4-phosphate. The red and turquoise modules were negatively correlated with glucose-1-phosphate, D-glucose-6-phosphate, D-fructose-6-phosphate, phosphoenolpyruvate, and D-erythrose-4-phosphate. These modules contained 19 sugar metabolism-related genes, with two, four, three, and ten genes assigned to the yellow, blue, red, and turquoise modules, respectively.

**Figure 5:**
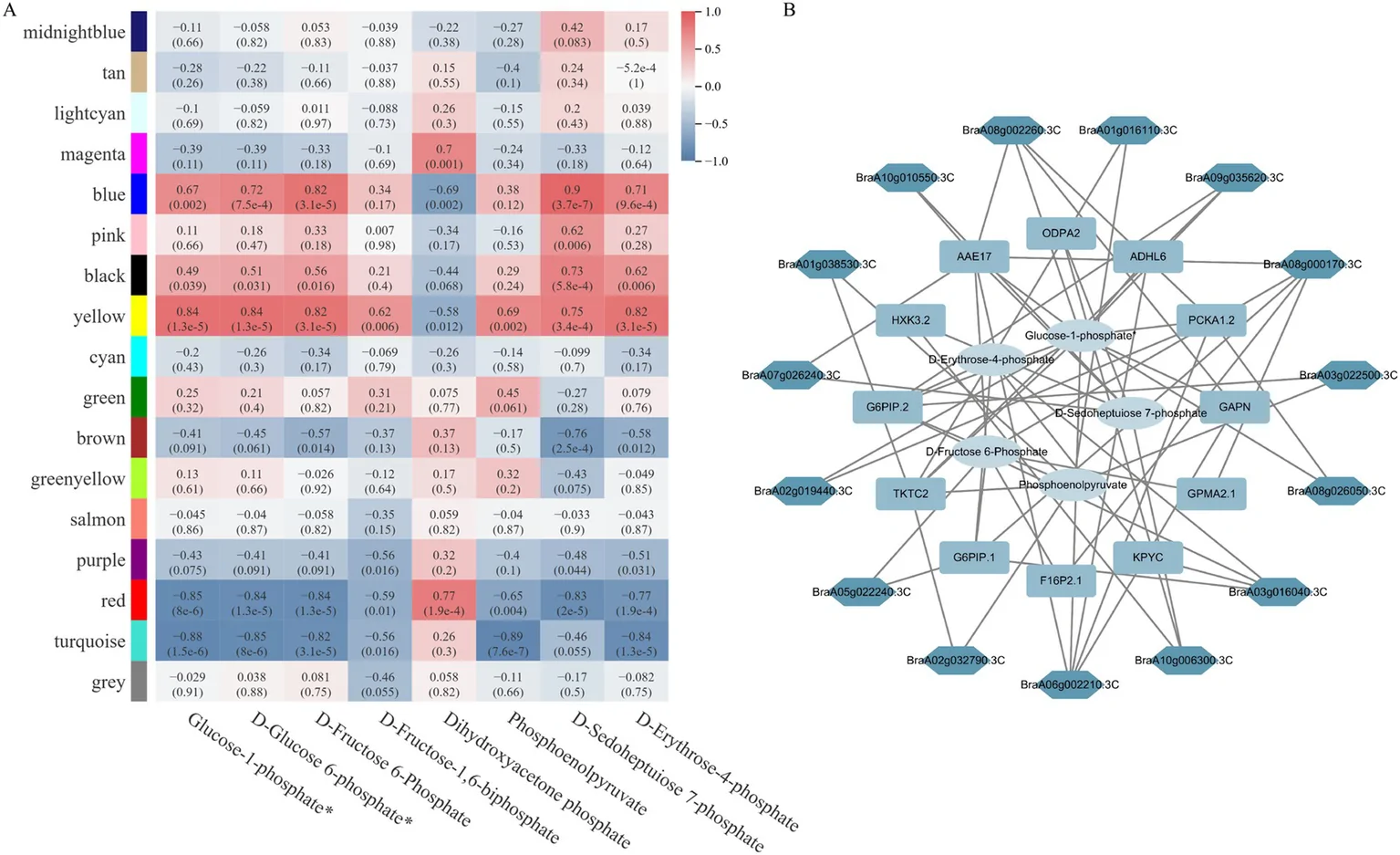
WGCNA-based association analysis of glycolysis- and gluconeogenesis-related sugar metabolism in Yushuyuangen. **(A)** Correlations between gene co-expression modules and differentially accumulated sugar metabolites mapped to the reconstructed glycolysis and gluconeogenesis pathways. **(B)** Co-expression network illustrating the relationships among transcription factors, structural genes, and key sugar metabolites involved in glycolysis and gluconeogenesis.

Expression correlation analysis between transcription factors from these four modules and structural genes in the reconstructed pathways identified 15 transcription factors correlated with 12 structural genes. These relationships were integrated with metabolite–gene correlations to construct a correlation network (Figure 5B). D-erythrose-4-phosphate, which accumulated preferentially in root flesh, was negatively correlated with *G6PIP.1*, *G6PIP.2*, and *LBD12* (*BraA03g016040.3C*), whereas both structural genes were positively correlated with *LBD12*. Similarly, glucose-1-phosphate was negatively correlated with *G6PIP.2*, *TKTC2*, and *WRKY65* (*BraA09g035620.3C*), while both structural genes were positively correlated with *WRKY65*. D-sedoheptulose-7-phosphate, which accumulated in YGW-SR, was positively correlated with a C2H2-type transcription factor (*BraA10g006300.3C*) and negatively correlated with *HXK3.2*; the transcription factor and *HXK3.2* were also negatively correlated. D-fructose-6-phosphate was positively correlated with *F16P2.1* (*r* = 0.96, *p* < 0.001) and *NAC22* (*BraA08g000170.3C*, *r* = 0.91, *p* < 0.001), but negatively correlated with *G6PIP.1* and *NAC16* (*BraA05g022240.3C*). *G6PIP.1* was positively correlated with *NAC16* and negatively correlated with *NAC22*, whereas *F16P2.1* was positively correlated with *NAC22*. Together, these associations highlighted candidate transcription factors linked to structural gene expression and the contrasting distributions of sugar metabolism intermediates among root tissues.

### 3.5 Integrated analysis of metabolites and gene expression in amino acid biosynthesis of Yushuyuangen

A total of 20 DAMs and 57 DEGs associated with amino acid biosynthesis were identified across five metabolic branches: aromatic amino acids, the serine family, the aspartate family, the glutamate family, and branched-chain amino acids (Figure 6). In the aromatic amino acid biosynthesis pathway, L-phenylalanine and L-tyrosine showed higher accumulation in YGW-SR and were negatively correlated with genes encoding 3-phosphoshikimate 1-carboxyvinyltransferase (*AROA.2*), anthranilate phosphoribosyltransferase (*TRPD.1*), and chloroplastic indole-3-glycerol phosphate synthase (*TRPC.1*). These genes generally showed higher expression in white peel and lower expression in root flesh. Similarly, L-tryptophan accumulated preferentially in YPW-SR and was negatively correlated with the gene encoding tryptophan synthase beta chain 2 (*TRBP2.2*, *r* = −0.82, *p* < 0.01), which showed relatively high expression in peel tissues and lower expression in root flesh. In the serine family amino acid pathway, L-serine accumulated at higher levels in YGW-SR and was negatively correlated with the gene encoding serine acetyltransferase 1 (*SAT1.1*), which showed low expression in this tissue. Within the aspartate family amino acid pathway, L-threonine, L-homoserine, and L-asparagine accumulated preferentially in root flesh. L-homoserine was negatively correlated with genes encoding 5-methyltetrahydropteroyltriglutamate–homocysteine methyltransferase (*METE1.1*, *METE1.2*, *METE2*, and *METE1.3*), which showed higher expression in white peel, particularly YGW-WP. In contrast, L-lysine accumulated at higher levels in purple peel (YPW-PP), while L-methionine showed higher accumulation in both green and white peel of YGW. Within the glutamate family amino acid pathway, L-glutamine and L-arginine showed higher accumulation in YPW-PP and YGW-WP, respectively. The branched-chain amino acids L-valine, L-leucine, and L-isoleucine shared higher accumulation in purple and green peel, although the associated structural genes exhibited distinct tissue expression patterns. For example, the gene encoding branched-chain amino acid aminotransferase (*BCAT2*) showed its highest expression in YGW-WP, and the gene encoding 3-isopropylmalate dehydratase small subunit 1 (*LEUD3*) was also preferentially expressed in YGW-WP.

**Figure 6:**
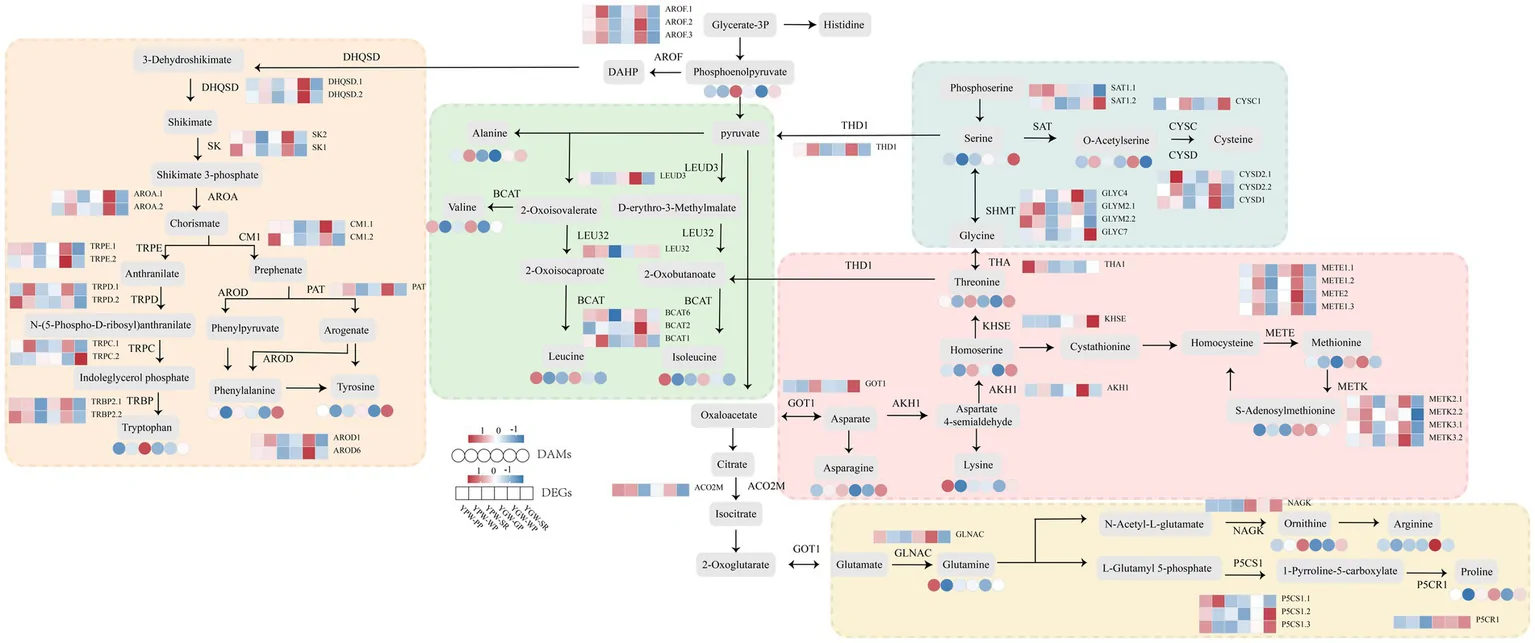
Integrated reconstruction of amino acid biosynthesis pathways in Yushuyuangen. Differentially expressed genes (DEGs) and differentially accumulated metabolites (DAMs) were mapped onto the pathways. Red and blue indicate up- and down-regulation, respectively.

WGCNA identified contrasting module–metabolite associations (Figure 7A). The yellow module was positively correlated with several amino acids showing higher accumulation in root flesh, including L-tryptophan, L-phenylalanine, L-threonine, L-homoserine, and L-asparagine, with relatively strong correlations observed for L-threonine, L-homoserine and L-tryptophan. The magenta module was positively correlated with L-methionine, S-(5′-adenosyl)-L-methionine, L-leucine, and L-isoleucine, including L-leucine (*r* = 0.71, *p* < 0.01) and L-methionine (*r* = 0.70, *p* < 0.01), while the tan module was positively correlated with the former two metabolites. Conversely, the turquoise and red modules were negatively correlated with several root-flesh-associated amino acids, including L-threonine and L-homoserine.

**Figure 7:**
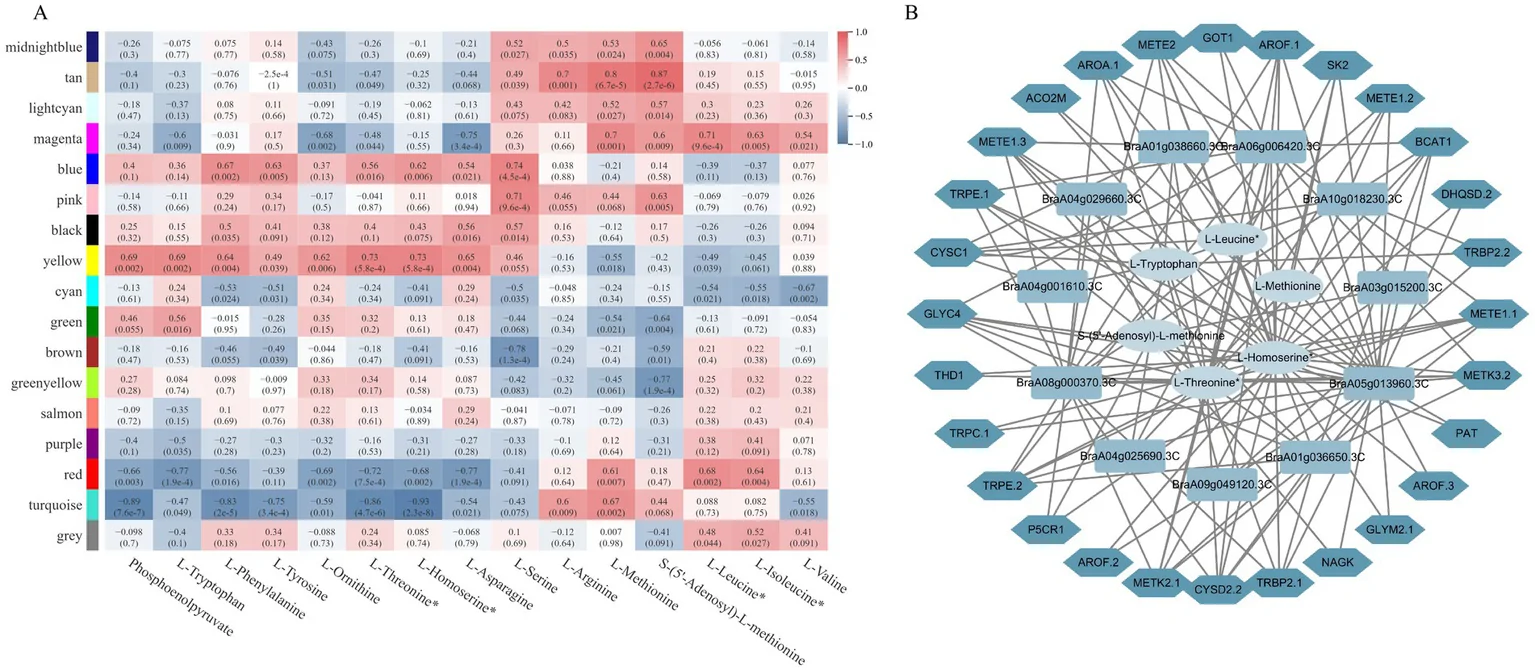
WGCNA-based association analysis of the amino acid biosynthesis pathways in Yushuyuangen. **(A)** Correlations between gene co-expression modules and metabolites mapped to the reconstructed amino acid biosynthesis pathways. **(B)** Correlation network showing relationships among transcription factors, structural genes, and key metabolites involved in amino acid biosynthesis.

Transcription factors from these five modules were examined for expression correlations with structural genes in the reconstructed pathways, including both module-associated genes and genes retrieved directly from transcriptome annotations. This analysis identified 11 transcription factors highly correlated with 28 structural genes; these relationships were integrated with metabolite–gene correlations to construct a correlation network (Figure 7B). L-tryptophan, which accumulated preferentially in YPW-SR, was negatively correlated with *bHLH65* (*BraA09g049120.3C*, *r* = −0.85, *p* < 0.01) and *ARFD4* (*BraA10g018230.3C*), both of which were positively correlated with *TRBP2.2*. L-homoserine, which accumulated preferentially in root flesh, was positively correlated with *LBD13* (*BraA05g013960.3C, r* = 0.816, *p* < 0.01). In contrast, the four *METE* genes were negatively correlated with both *LBD13* and L-homoserine. These associations highlighted candidate transcription factors linked to structural gene expression and tissue-dependent amino acid accumulation.

### 3.6 Integrated analysis of metabolites and gene expression in phenylpropanoid metabolism of Yushuyuangen

Phenolic acids and flavonoids were analyzed as two major groups of phenylpropanoid-related metabolites to characterize their tissue-specific accumulation and associations with structural genes and candidate transcription factors.

#### 3.6.1 Integrated analysis of metabolites and gene expression in phenolic acid-related phenylpropanoid metabolism of Yushuyuangen

A total of 11 DAMs and 34 DEGs associated with phenolic acid-related phenylpropanoid metabolism were identified (Figure 8). Colored peel showed distinct accumulation patterns of phenylpropanoid-related metabolites. Cinnamic acid, *p-*coumaric acid, and ferulic acid accumulated at higher levels in YPW-PP, whereas sinapic acid showed higher accumulation in both purple and green peel. *p-*coumaric acid was positively correlated with genes encoding phenylalanine ammonia-lyase (*PAL1.2*) and trans-cinnamate 4-monooxygenase (*C4H.2*), although both genes showed higher expression in peel tissues than in root flesh, with *PAL1.2* most highly expressed in YPW-WP and *C4H.2* in YGW-WP. It was negatively correlated with the gene encoding cinnamoyl-CoA reductase (*CCR35*), which showed high expression in YGW-SR. YGW-GP was characterized by higher accumulation of coniferin and syringin. Syringin was positively correlated with *CCR1.4* (*r* = 0.88, *p* < 0.01) and the gene encoding cinnamyl alcohol dehydrogenase (*CADH7.1*), with the latter also showing higher expression in YGW-GP. White peel showed higher accumulation of *p-*coumaraldehyde, which was positively correlated with *CCR1.4* and *CADH7.2* (*r* = 0.98, *p* < 0.01). *CCR1.4* showed higher expression in white peel, particularly YPW-WP, whereas *CADH7.2* showed higher expression in both green and white peel of YGW. In contrast, root flesh showed lower accumulation of coniferin and syringin, while the upstream precursor L-phenylalanine accumulated at higher levels in YGW-SR. L-phenylalanine was negatively correlated with *PAL2.2*, which showed higher expression in white peel and lower expression in root flesh (Figure 8).

**Figure 8:**
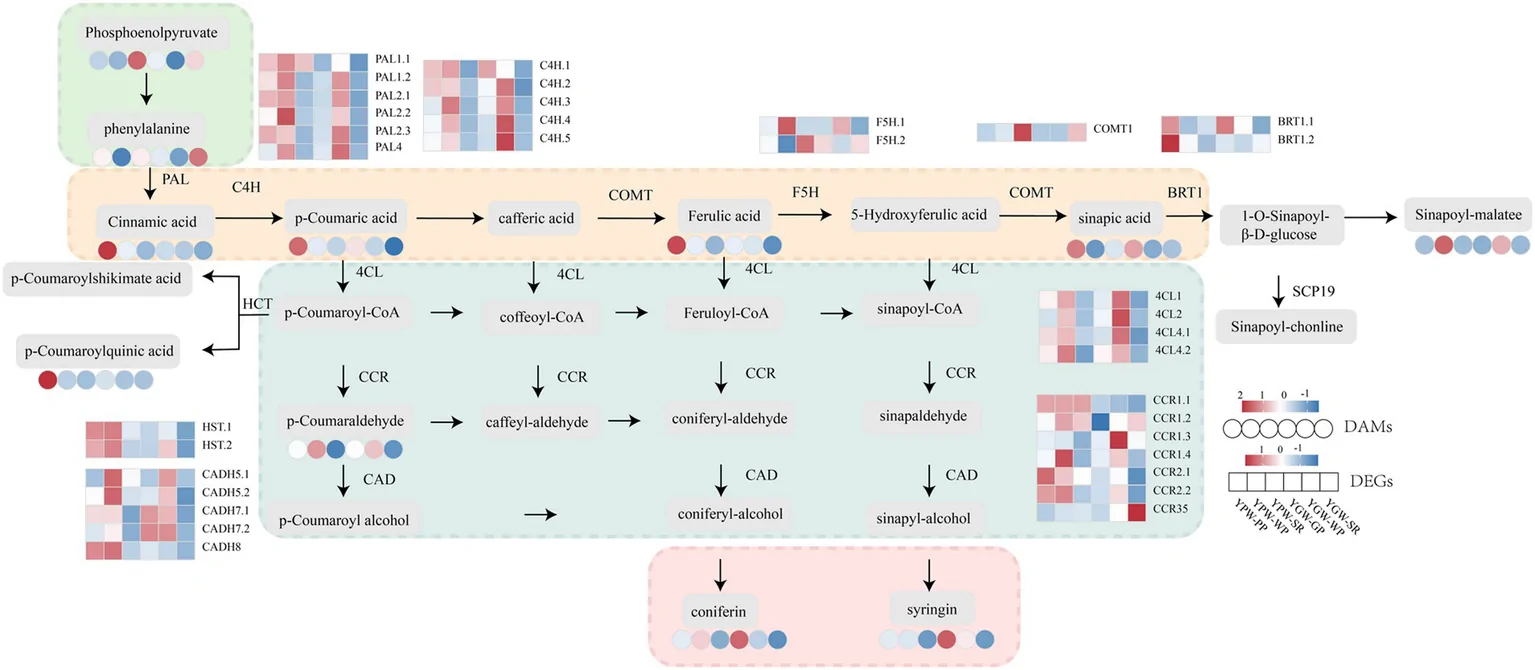
Integrated reconstruction of the phenylpropanoid pathway related to phenolic acid biosynthesis in Yushuyuangen. Differentially expressed genes (DEGs) and differentially accumulated metabolites (DAMs) were mapped onto the pathway. Red and blue indicate up- and down-regulation, respectively.

WGCNA of phenylpropanoid-related metabolites revealed significant associations between multiple co-expression modules and these metabolites (Figure 9A). The red module was positively correlated with cinnamic acid (*r* = 0.64, *p* < 0.01), *p-*coumaric acid (*r* = 0.78, *p* < 0.01), *p-*coumaraldehyde (*r* = 0.70, *p* < 0.01), ferulic acid, syringin, and coniferin, encompassing metabolites with different accumulation patterns among peel tissues. The blue and yellow modules were negatively correlated with *p-*coumaric acid, *p-*coumaraldehyde, and ferulic acid.

**Figure 9:**
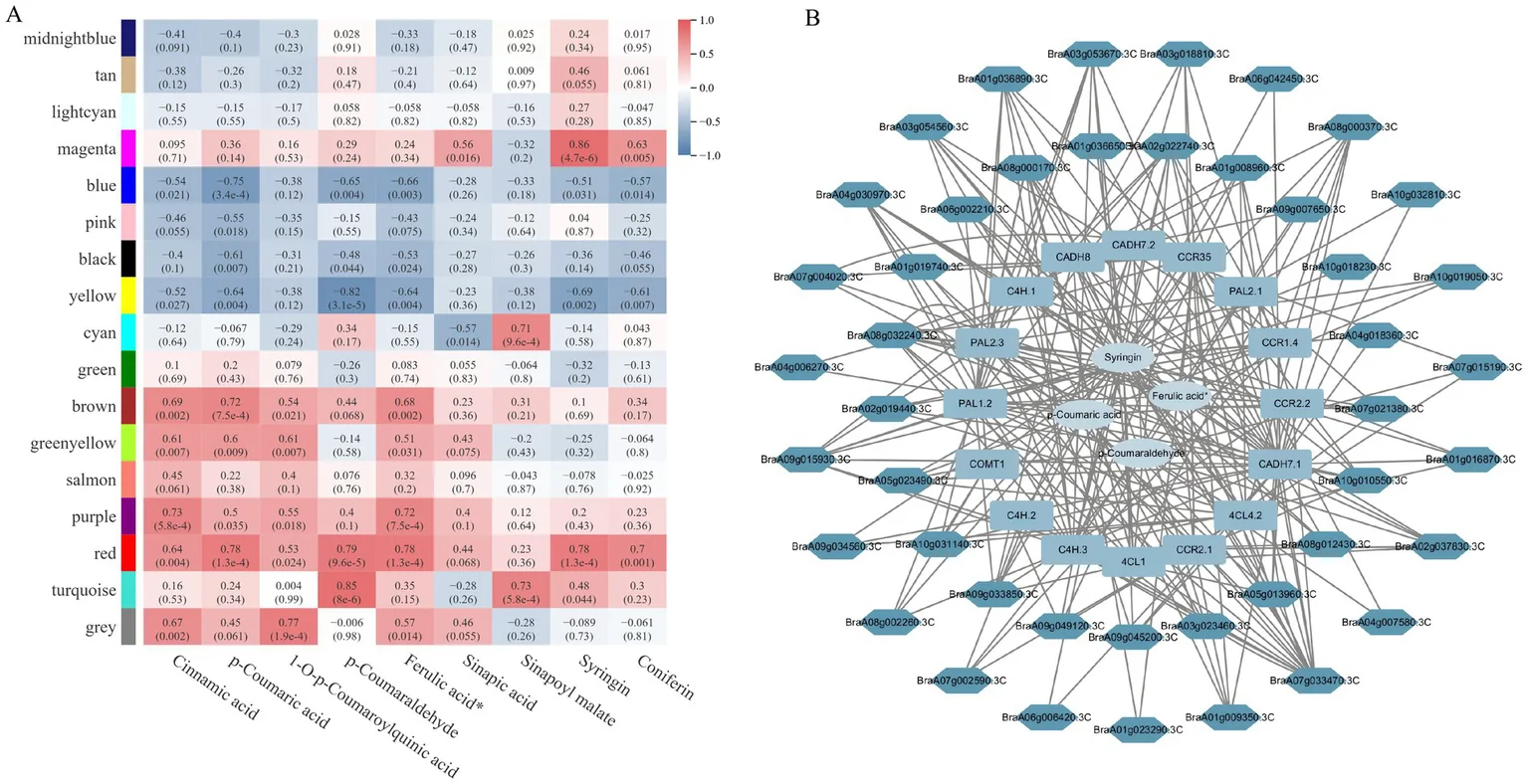
WGCNA-based association analysis of the phenylpropanoid pathway related to phenolic acid biosynthesis in Yushuyuangen. **(A)** Correlations between gene co-expression modules and metabolites mapped to the reconstructed phenylpropanoid pathway related to phenolic acid biosynthesis. **(B)** Correlation network showing relationships among transcription factors, structural genes, and key metabolites involved in the phenylpropanoid pathway.

Expression correlation analysis between transcription factors from these three modules and structural genes in the reconstructed pathway identified 44 transcription factors highly correlated with 16 structural genes. These relationships were integrated with metabolite–gene correlations to construct a correlation network (Figure 9B). Within this network, *p-*coumaric acid, which accumulated preferentially in YPW-PP, was positively correlated with *PAL1.2* (*r* = 0.80, *p* < 0.01) and *C4H.2* (*r* = 0.80, *p* < 0.01) and negatively correlated with *CCR35*. It was also positively correlated with *MYB62* (*BraA07g033470.3C*; *r* = 0.88, *p* < 0.01), *AGL63* (*BraA09g033850.3C*), and *ERF25* (*BraA10g010550.3C*). *MYB62* and *AGL63* were positively correlated with *PAL1.2* and *C4H.2* and negatively correlated with *CCR35*. Similarly, *ERF25* was positively correlated with *PAL1.2* but negatively correlated with *CCR35*. *p-*Coumaraldehyde, which accumulated at higher levels in white peel, was positively correlated with *CCR1.4* and *CADH7.2*. It was also positively correlated with *BZIP2* (*BraA07g002590.3C*), *LOB* (*BraA09g007650.3C*), and *bHLH65* (*BraA09g049120.3C*), but negatively correlated with *SCL1* (*BraA07g015190.3C*). *BZIP2* was positively correlated with both *CCR1.4* and *CADH7.2*. In addition, *CADH7.2* was positively correlated with *LOB* and *bHLH65* and negatively correlated with *SCL1*.

#### 3.6.2 Integrated analysis of metabolites and gene expression in flavonoid biosynthesis of Yushuyuangen

A total of 81 DAMs and eight DEGs were associated with flavonoid biosynthesis (Figure 10). The DAMs comprised six groups: flavonols, flavones, flavanones, flavanols, chalcones, and flavanonols (Figure 10). Genes involved in flavonoid biosynthesis showed clear tissue-specific expression patterns. Genes encoding chalcone isomerase-like proteins (*CFI* and *CFI3*) and flavonol synthases (*FLS*, *FLS1*, and *FLS3*) generally showed higher expression in peel tissues than in root flesh, whereas the three flavonoid 3′-monooxygenase genes (*F3′H1.1*, *F3′H1.2*, and *F3′H1.3*) showed particularly high expression in YGW-WP. Consistent with these expression patterns, flavonols, flavanones, and flavones were also predominantly accumulated in peel tissues, especially in the colored peel of YPW and YGW. Among these, *FLS1* was positively correlated with the flavonols kaempferol-3-*O*-galactoside (*r* = 0.91, *p* < 0.01) and kaempferol-7-*O*-glucoside (*r* = 0.90, *p* < 0.01), as well as pinocembrin-7-*O*-(6″-*O*-malonyl)glucoside (*r* = 0.94, *p* < 0.01). *F3′H1.2* was likewise positively correlated with trifolin (*r* = 0.84, *p* < 0.01) and pinocembrin-7-*O*-(6″-*O*-malonyl)glucoside (*r* = 0.89, *p* < 0.01).

**Figure 10:**
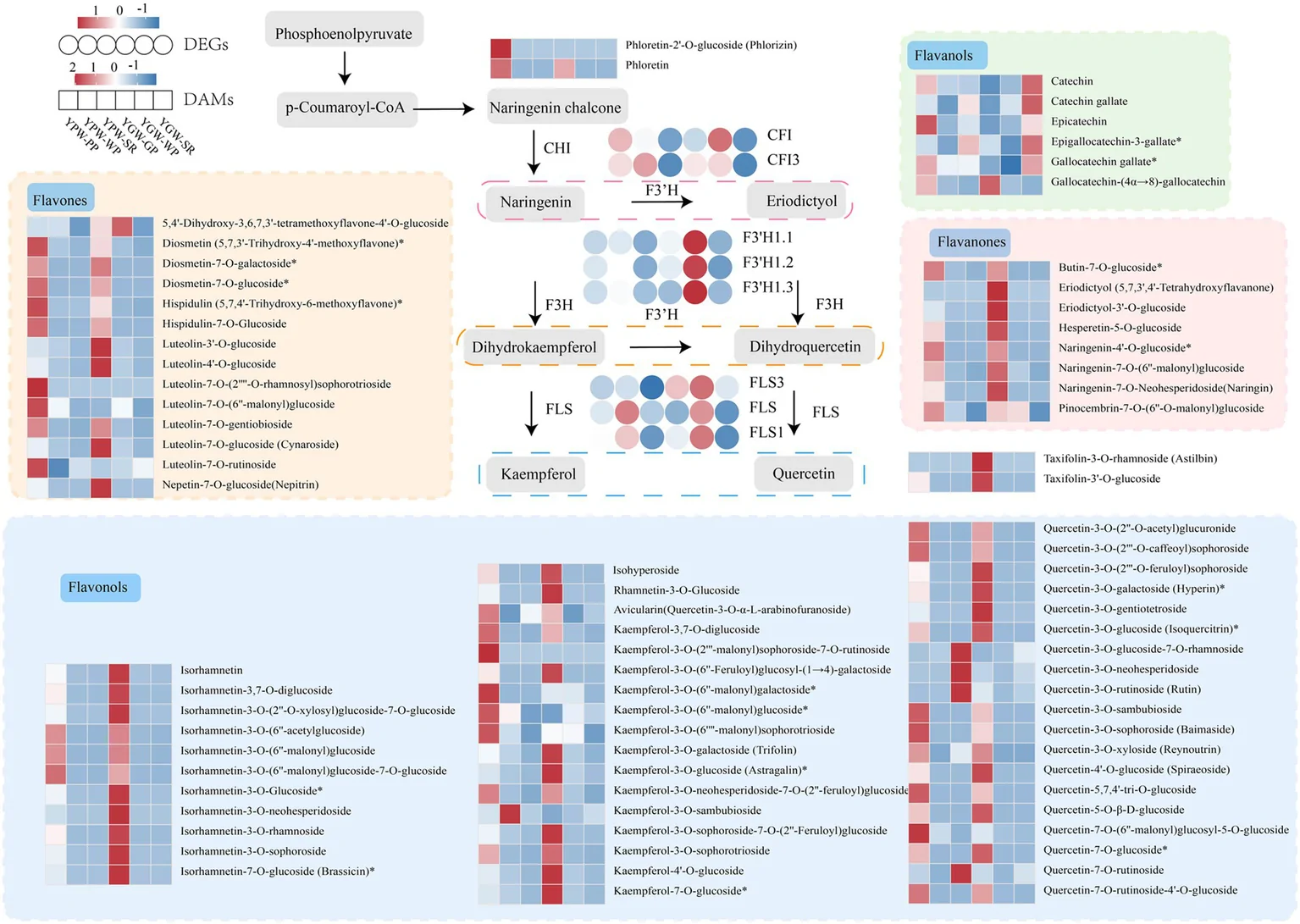
Integrated reconstruction of the phenylpropanoid pathway related to flavonoid biosynthesis in Yushuyuangen. Differentially expressed genes (DEGs) and differentially accumulated flavonoids were mapped onto the pathway. Red and blue indicate up- and down-regulation, respectively.

WGCNA identified the magenta and red modules as positively associated with many flavonoids (Supplementary Figure S7). Two flavonoid biosynthesis-related structural genes were identified within these flavonoid-associated modules. Expression correlation analysis was subsequently performed between transcription factors from these modules and structural genes in the reconstructed flavonoid pathway. This analysis identified six transcription factors highly correlated with four structural genes, and these relationships were integrated with metabolite–gene correlations to construct a correlation network (Figure 11).

**Figure 11:**
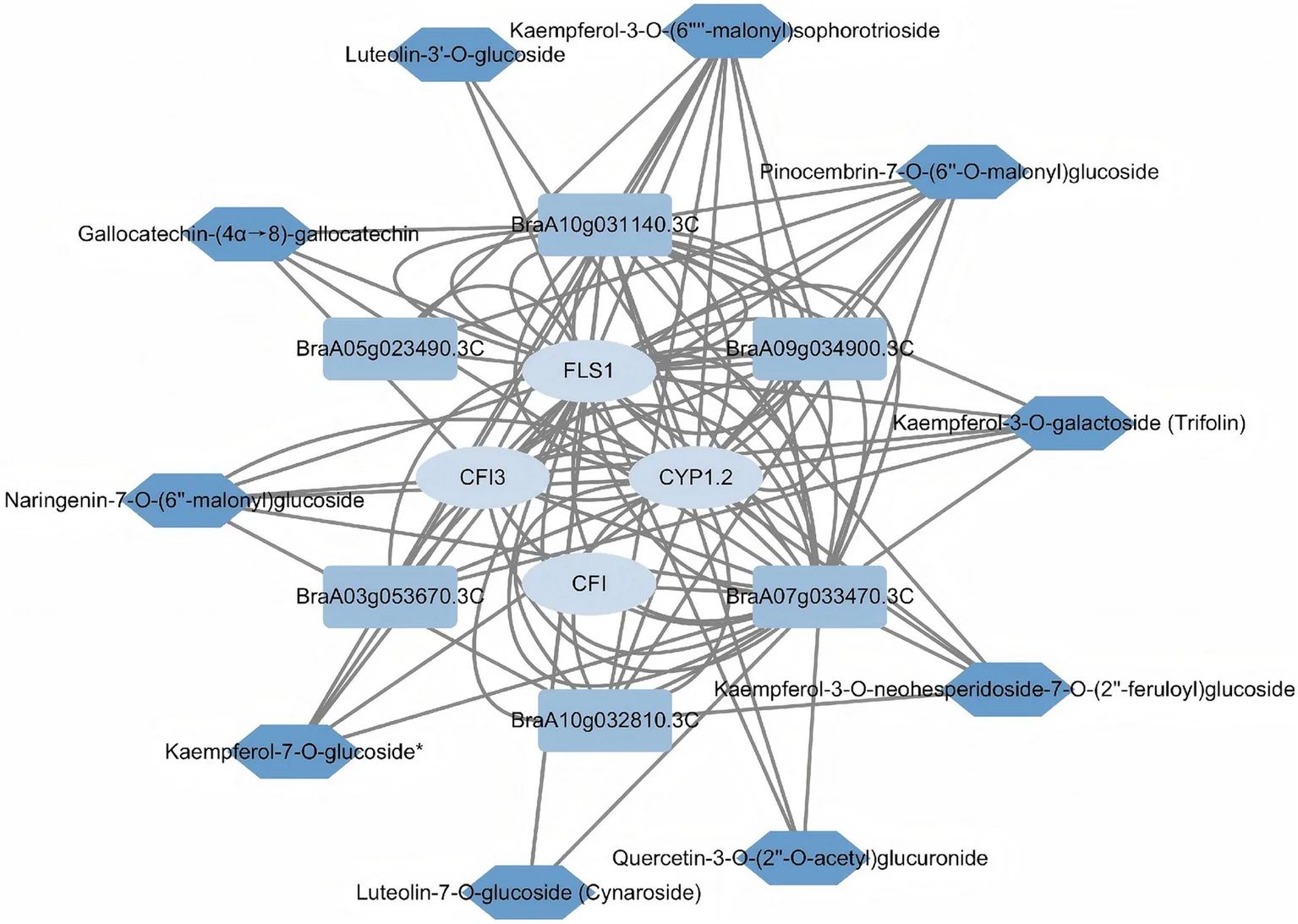
Correlation network showing relationships among transcription factors, structural genes, and key metabolites involved in the phenylpropanoid pathway related to flavonoid biosynthesis in Yushuyuangen.

Within this network, luteolin-3′-*O*-glucoside, which accumulated preferentially in YGW-GP, was positively correlated with *FLS1* (*r* = 0.81, *p* < 0.01) and *MYB*62 (*BraA07g033470.3C*, *r* = 0.87, *p* < 0.01). Kaempferol-3-*O*-galactoside (trifolin) and kaempferol-7-*O*-glucoside, which also showed higher accumulation in YGW-GP, were positively correlated with *FLS1*, *F3′H1.2*, and the same MYB transcription factor. Pinocembrin-7-*O*-(6″-*O*-malonyl)glucoside and kaempferol-3-*O*-(6‴‴-malonyl)sophorotrioside, which showed higher accumulation in YPW-PP, were strongly correlated with *FLS1*, *F3′H1.2*, and *NAC82* (*BraA10g031140.3C*). These associations highlighted structural genes and transcription factors linked to the distinct flavonoid accumulation patterns of purple and green peel.

## 4 Discussion

Through the combined analysis of metabolome and transcriptome, this study systematically compared the quality-related metabolism between green-white (YGW) and purple-white (YPW) Yushuyuangen roots in different tissues. The results revealed clear tissue-specific patterns in metabolite accumulation, mainly involving four major categories: amino acids, flavonoids, phenolic acids, and carbohydrates. In general, representative sugars and amino acids were mainly enriched in root flesh, whereas phenolic acids and flavonoids were accumulated in colored peel tissues. Differences in metabolic patterns between varieties within corresponding tissues indicate that genetic background also contributes to variation in quality-related metabolism.

### 4.1 Influence of carbohydrate metabolism on flavor

Several phosphorylated sugar intermediates, including G1P, G6P, F6P, E4P, and S7P, accumulated preferentially in Yushuyuangen root flesh. Similar accumulation of G1P, G6P, and F6P has been reported in potato tubers (25). Consistent with these metabolic patterns, we identified several genes involved in hexose-phosphate metabolism, including *HXK* and glucose-6-phosphate isomerase genes, which have also been identified in radish fleshy roots (26). E4P has been reported to inhibit phosphoglucose isomerase (*G6PI/PGI*) activity (27), while *G6PI* catalyzes the reversible interconversion of G6P and F6P. These known biochemical relationships are consistent with the associations observed in the present study. In addition, in Arabidopsis (28), *WRKY18* and *WRKY53* directly bind to W-box elements in the *GPT2* promoter and activate its expression, while a *CsWRKY29*–*CsABI5*–*CsHXK1/CsSUS4* regulatory cascade has been reported in tea plant (29). These findings support a role for WRKY transcription factors in the direct or indirect regulation of genes associated with sugar-phosphate and hexose metabolism. Thus, the observed associations of *WRKY65* with *G6PIP.2* and *TKTC2* suggest its potential involvement in carbohydrate metabolism in Yushuyuangen.

### 4.2 Amino acid metabolism and flavor formation

Amino acid composition differed markedly among Yushuyuangen root tissues. Aromatic amino acids and several members of the aspartate family accumulated preferentially in root flesh, whereas the branched-chain amino acids (BCAAs) valine, leucine, and isoleucine were more abundant in colored peel. Similar tissue-level metabolic partitioning has been reported in other storage organs; for example, potato tuber flesh preferentially accumulates primary metabolites such as amino acids, whereas the skin is enriched in defense-related secondary metabolites (13). Previous studies have mainly reported genotype-dependent variation in amino acid accumulation among root and tuber crops. For example, metabolomic analyses of radish have shown pronounced differences in amino acid profiles among fleshy-root genotypes (30). In contrast, our results further indicate that the differences between Yushuyuangen peel and root flesh do not represent uniform changes across all amino acids, but instead show clear pathway-specific patterns. Because aromatic amino acids and BCAAs serve as important precursors for diverse volatile compounds, their selective accumulation may contribute to differences in both nutritional composition and the availability of flavor precursors among tissues (31).

In the present study, aromatic amino acid accumulation did not closely parallel the expression of associated structural genes; notably, *TRPD.1* was negatively correlated with phenylalanine and tyrosine. Because tryptophan, phenylalanine and tyrosine biosynthesis diverge from the common precursor chorismate, previous studies have shown that carbon flux can be differentially allocated among these competing aromatic amino acid branches (32, 33). Thus, although *TRPD.1* functions in the tryptophan biosynthetic branch, its negative association with phenylalanine and tyrosine may reflect differential metabolic allocation downstream of chorismate rather than a direct inhibitory effect on their accumulation. Meanwhile, *bHLH65* and *ARFD4* were both positively correlated with *TRBP2.2* but negatively correlated with tryptophan. In Arabidopsis, bHLH factors such as *ATR2* and *MYC2* have been implicated in the transcriptional regulation of tryptophan biosynthesis and tryptophan-related metabolism (34, 35), providing family-level functional support for the involvement of *bHLH65*. Accordingly, the *bHLH65–TRBP2.2–*tryptophan correlation pattern suggests a potential association of *bHLH65* with tissue-specific tryptophan branch, whereas *ARFD4* represents an additional candidate factor requiring further validation.

### 4.3 Phenylpropanoid acid metabolism

Phenylpropanoid accumulation showed clear tissue specificity in Yushuyuangen roots, with distinct phenolic acid and flavonoid profiles among colored peel, white peel, and root flesh. Similar tissue- and genotype-dependent variation has been reported in turnip, including differences in cinnamic, p-coumaric, and ferulic acids and several major flavonoids between peel and pulp tissues (36). These findings support the tissue-specific distribution of phenylpropanoid metabolites observed in Yushuyuangen.

Integrated analysis revealed tissue-specific associations among phenylpropanoid metabolites, structural genes, and transcription factors. *p-*coumaric acid was positively correlated with *PAL1.2* and *C4H.2*, and the correlation network further linked these components with *MYB62*, *AGL63*, and *ERF25*. Previous studies have shown that R2R3-MYB transcription factors can regulate upstream phenylpropanoid genes. Heterologous overexpression of *LjMYB106* was accompanied by increased *PAL* and *C4H* expression and enhanced *p-*coumaric acid accumulation (37), whereas *VvMYB14* directly activated *PAL* and *C4H* transcription in grapevine (38). These findings provide functional context for the observed association of *MYB62* with the *PAL*/*C4H*–*p-*coumaric acid module in Yushuyuangen, although this association requires further experimental validation.

Flavonoid accumulation in Yushuyuangen showed clear tissue specificity, and integrated correlation analysis identified close associations among flavonoid derivatives, structural genes, and candidate transcription factors. *FLS1* was positively correlated with trifolin, kaempferol-7-*O*-glucoside, and kaempferol-3-*O*-(6″″-malonyl)sophorotrioside. Functional studies in Brassica species have confirmed the role of *FLS* in flavonol formation (39, 40). In *Brassica rapa* subsp. *chinensis*, *BcMYB111* directly binds the promoters of *BcF3H* and *BcFLS1* and promotes flavonol accumulation (41), providing closely related functional context for the *MYB62*–*FLS1*/*F3′H1.2*–flavonol association identified here. BraA10g031140.3C was also associated with *FLS1*, *F3′H1.2*, and several flavonoid derivatives. These transcription factors therefore represent candidate factors associated with flavonoid accumulation, whose specific functions require further experimental validation. In addition, some flavonoid metabolites and associated structural genes showed incomplete tissue-level synchronization, indicating that transcript abundance alone may not fully explain metabolite accumulation.

## 5 Conclusion

Integrated metabolomic and transcriptomic analyses revealed clear tissue- and variety-dependent metabolic differentiation in Yushuyuangen roots, involving carbohydrate, amino acid, phenylpropanoid, and flavonoid metabolism. Root flesh was characterized mainly by the accumulation of sugar-phosphate intermediates and several amino acids, whereas colored peel showed greater accumulation of phenolic acids, flavonoids, and branched-chain amino acids. Integrated WGCNA and correlation analyses further identified tissue-associated relationships among metabolites, structural genes, and transcription factors. Four candidate transcription factor–structural gene–metabolite associations were highlighted: *NAC22*–*F16P2.1*–D-fructose-6-phosphate, *bHLH65*/*ARFD4*–*TRBP2.2*–L-tryptophan, *MYB62*/*AGL63*–*PAL1.2*/*C4H.2*–*p-*coumaric acid, and *MYB62*–*FLS1*/*F3′H1.2*–trifolin/kaempferol-7-*O*-glucoside. These associations were inferred from co-expression and correlation patterns and represent candidate relationships for further functional validation.

## Data Availability

The RNA-seq data have been deposited in the NCBI Sequence Read Archive (SRA) under BioProject accession PRJNA1378371.

## Supplementary material

SUPPLEMENTARY FIGURE S1 Different storage-root tissues of the two Yushuyuangen varieties.

SUPPLEMENTARY FIGURE S2 Permutation test results of the OPLS-DA models for the nine pairwise comparisons.

SUPPLEMENTARY FIGURE S3 Evaluation of UPLC–MS/MS data stability and reliability. **(A)** Coefficient of variation (CV) distribution of all detected metabolites. **(B)** Total ion chromatogram (TIC) of N samples. **(C)** Total ion chromatogram (TIC) of P samples.

SUPPLEMENTARY FIGURE S4 Differentially accumulated metabolites with the largest fold changes in six pairwise comparisons.

SUPPLEMENTARY FIGURE S5 KEGG co-enrichment analysis of transcriptomic and metabolomic data.

SUPPLEMENTARY FIGURE S6 Selection of the soft-thresholding power in WGCNA.

SUPPLEMENTARY FIGURE S7 WGCNA module analysis associated with flavonoid metabolism.

SUPPLEMENTARY TABLE S1 Qualitative and quantitative metabolomic data of all samples analyzed by UPLC-MS/MS.

SUPPLEMENTARY TABLE S2 Qualitative and quantitative transcriptomic data of differentially expressed transcription factors in all analyzed samples.
